# Household Hints and Recipes

**Published:** 1880-10

**Authors:** 


### Household Hints & Recipes

INK STAINS are best removed from linen by means of a solution of oxolic and citric acids in about equal quantities, the goods being thoroughly rinsed in water immediately afterwards.

---

RANCID BUTTER may be improved by kneading it well with twenty per cent. lime water for about five minutes ; then the water is poured off, the butter washed with clear water, and salted anew.

---

MOUTH WASH.—The following is a good mixture to strengthen the gums :

Alcohol, 20 troy ounces.
Chloroform, 1 to 2 " "
Oil of peppermint, sufficient.

---

GOLD VARNISH FOR METALS.—Dr. Taylor found picric acid and boracic acid in a gold varnish for metals, which afforded a very hard and beautiful surface; and he recommends a clear solution of shellac, with the addition of picric acid, and about half of one per cent. of boracic acid, as giving results equally good.

---

CHICKEN CHOLERA.—The following has recently been recommended :

Sulphuric acid, 1 fluid ounce.
Sulphate of iron, 1 pound.
Water, sufficient to make 1 gallon.

The dose is one ounce of the mixture to a pint of water, which is to be given to drink in place of ordinary water, or mixed with the food served to the fowls.

---

BREAD AS A DIGESTIVE AGENT.—Scheurer-Kestner, in *Pharm. Centralh.*, has found that in fermenting dough a substance is formed which digests meat perfectly. "Meat-bread" is prepared as follows : Eleven to twelve ounces of flour are mixed with one ounce of baker's yeast, and six ounces of finely chopped meat : sufficient water is added to make a suitable dough, and the mixture is set aside for two or three days in a warm place ; the dough is then completed, and baked as usual. This is a very nutritious aliment, the taste of which can be improved by substituting for a part of the raw meat some bacon, and adding onions, spices, etc.

WATERPROOFING BOOTS.--One quart of boiled linseed oil, four ounces of mutton suet, one ounce and a half of beeswax, and one ounce of resin are melted together, and brushed on the dry boots while still hot.

---

TO AGE OAK FURNITURE.—Place the furniture in a small, dark room ; put on the floor a dish or saucer containing water of ammonia, and leave the furniture there till the color is sufficiently darkened. This method works better than washing the furniture with diluted ammonia.

---

CLOTHES WRINGER AS PRESS.—Dieterich recommends, in *Droguisten-Zeit.*, the use of the ordinary clothes wringer as a pharamaceutical press. He even uses it for plasters and ointments, but for this latter purpose wraps around the press-bag, or cloth, a piece of parchment paper, which prevents the rubber rollers of the wringer from being soiled.

---

IMPROVED POLISHING CLOTH.—The ordinary polishing cloth, consisting of coarse linen or hemp fabric impregnated with emery or tripoli by means of glue or paste, cannot be used for wet polishing. It is recommended to use chromated glue as an adhesive material, and then expose it to sunlight, which renders the glue insoluble even in hot water.

---

COLORED INKS.—If you do not like aniline inks, you may prepare good colored ink by the following formulæ, some of which we have published before:

*Green.*—Two parts acetate of copper, one part carbonate of potassium, and eight parts water. Boil till half evaporated, and filter.

*Blue.*—Three parts Prussian blue, one part oxalic acid, and thirty parts of water, when dissolved add one part of gum arabic.

*Yellow.*—One part of fine orpiment well rubbed up with four parts of thick gum-water.

*Red.*—Dissolve four grains of carmine by trituration in one ounce of water of ammonia, and add six grains of gum-arabic.

*Gold.*—Rub gold-leaf, such as is used by bookbinders, with honey till it forms a uniform mixture. When the honey has been washed out with water, the gold powder will settle at the bottom, and must be mixed with gum-water in sufficient quantity.

*Silver.*—Silver-leaf treated in the same manner gives a silver ink. Both these inks may, when dry, be polished with ivory.

*Black.*—Three ounces of crushed nutgalls, two ounces of sulphate of iron, two ounces of gum arabic, and twenty-four ounces of water.

*White.*—Fine French zinc white, or white lead, rubbed up with gum-water to the proper consistency.

---

TO RENDER FABRIC INCOMBUSTIBLE it is recommended to soak it in a five per cent. solution of phosphate of ammonia. The fabric can only be charred, but will not burn with flame. If to the above phosphate solution five per cent. of alum be added, it will even be impossible to char the tissue.

---

TO PREVENT GLUE FROM CRACKING.—The cracking of glue, which frequently occurs when glued objects become very dry or are subjected to the heat of a stove, it is said, may be prevented by the addition of chloride of calcium to the glue, which prevents its drying so completely as to become brittle. Glue thus treated will adhere to glass, metals, etc., and can be employed for affixing labels to bottles.

---

LIQUID GLUE.—100 parts of ordinary gelatin are dissolved in 400 parts of water containing 6 to 7 parts of oxalic acid. The solution is kept for 5 or 6 hours on the water-bath, in a porcelain infusion pot, after which it is neutralized with carbonate of calcium, the insoluble precipitate filtered off, and the clear filtrate evaporated at a moderate temperature, until about 200 parts are obtained. The product is a durable, slightly tinted, but clear liquid glue.—*Pharm. Central-Anz.*

---

MARKING–INK FOR LINEN, ETC.—Moisten the place first with a solution of soda and gum-arabic; then iron it with a warm sad-iron, and write upon it with a goose-quill dipped into a solution of 1 part of chloride of platinum in 8 parts of water. When the writing is perfectly dry, it is traced over again with a solution of 1 part of stannous chloride in 16 parts of water. The writing then immediately assumes a brillant purple color.—*Pharm. Zeit. f. Russl.*

THE BLACK BRICKS now employed in the ornamentation of buildings are prepared by dipping in coal tar, the quality of bricks taken being the same as those used in other parts of the building. Black mortar for fancy joints is made by mixing with lamp-black.

---

CEMENT FOR RUBBER.—Powdered shellac is softened in ten times its weight of strong water of ammonia, whereby a transparent mass is obtained, which becomes fluid after keeping some little time without the use of hot water. In three or four weeks the mixture is perfectly liquid, and, when applied, it will be found to soften the rubber. As soon as the ammonia evaporates, the rubber hardens again—it is said quite firmly—and thus becomes impervious both to gases and to liquids. For cementing sheet rubber or rubber material in any shape, to metal, glass, and other smooth surfaces, the cement is highly recommended.

---

CLEANING OF PAINT BRUSHES.—Old paint may be removed from paint-brushes, even after they have become completely hard, by the following method: dissolve 1 lb. of caustic soda in 6 quarts of water, hang the brushes into the solution contained in a suitable vessel, so that they do not touch the bottom, and heat it for 12 to 24 hours to a temperature of 50° to 55° R. (122° to 131° F.) The dry paint thereby becomes so much softened that it may be completely washed out with soap and water. The temperature must not be allowed to exceed 55° C. (131° F.), since otherwise the hairs of the brush would be attacked by the soda.—*Phil. Notizbl.* and *Pharm. Z. f. Russl.*

---

ARTIFICIAL VANILLIN FROM OIL OF CLOVES.—For this purpose oil of cloves is diluted with three times its bulk of ether, and the liquid is shaken with a very diluted aqueous solution of potassa. An alkaline solution of eugenol is formed, which is separated from the oil, acidulated, and shaken with ether, which dissolved the eugenol. The ether being distilled off, the eugenol is treated with anhydrous acetic acid, and the aceto-eugenol thus formed is, in a very dilute solution, oxidized with a very weak, warm solution of permanganate of potassa. The product is then filtered, rendered slightly alkaline, and concentrated. Lastly, it is acidulated and shaken with ether, to extract the vanillin.

